# Exosomal annexin A6 induces gemcitabine resistance by inhibiting ubiquitination and degradation of EGFR in triple-negative breast cancer

**DOI:** 10.1038/s41419-021-03963-7

**Published:** 2021-07-08

**Authors:** Ting Li, Zhonghua Tao, Yihui Zhu, Xiaojia Liu, Leiping Wang, Yiqun Du, Jun Cao, Biyun Wang, Jian Zhang, Xichun Hu

**Affiliations:** 1 Department of Medical Oncology, Fudan University Shanghai Cancer Center, 200032 Shanghai, China; 2 Department of Oncology, Shanghai Medical College, Fudan University, 200032 Shanghai, China; 3 Division of Surgical Pathology, Huashan Hospital, Fudan University, 200040 Shanghai, China

**Keywords:** Breast cancer, Predictive markers

## Abstract

Exosomes are carriers of intercellular information that regulate the tumor microenvironment, and they have an essential role in drug resistance through various mechanisms such as transporting RNA molecules and proteins. Nevertheless, their effects on gemcitabine resistance in triple-negative breast cancer (TNBC) are unclear. In the present study, we examined the effects of exosomes on TNBC cell viability, colony formation, apoptosis, and annexin A6 (ANXA6)/EGFR expression. We addressed their roles in gemcitabine resistance and the underlying mechanism. Our results revealed that exosomes derived from resistant cancer cells improved cell viability and colony formation and inhibited apoptosis in sensitive cancer cells. The underlying mechanism included the transfer of exosomal ANXA6 from resistant cancer cells to sensitive cancer cells. Isobaric peptide labeling–liquid chromatography–tandem mass spectrometry and western blotting revealed that ANXA6 was upregulated in resistant cancer cells and their derived exosomes. Sensitive cancer cells exhibited resistance with increased viability and colony formation and decreased apoptosis when ANXA6 was stably overexpressed. On the contrary, knockdown ANXA6 restored the sensitivity of cells to gemcitabine. Co-immunoprecipitation expression and GST pulldown assay demonstrated that exosomal ANXA6 and EGFR could interact with each other and exosomal ANXA6 was associated with the suppression of EGFR ubiquitination and downregulation. While adding lapatinib reversed gemcitabine resistance induced by exosomal ANXA6. Moreover, ANXA6 and EGFR protein expression was correlated in TNBC tissues, and exosomal ANXA6 levels at baseline were lower in patients with highly sensitive TNBC than those with resistant TNBC when treated with first-line gemcitabine-based chemotherapy. In conclusion, resistant cancer cell-derived exosomes induced gemcitabine resistance via exosomal ANXA6, which was associated with the inhibition of EGFR ubiquitination and degradation. Exosomal ANXA6 levels in the serum of patients with TNBC might be predictive of the response to gemcitabine-based chemotherapy.

## Introduction

Breast cancer, especially triple-negative breast cancer (TNBC), is the most common malignancy in women and a serious threat to public health. Patients with TNBC cannot be treated with endocrine therapy or anti–HER-2 targeted therapies because of the absence of relevant receptors [1]. Recent efforts have led to the development of new therapies that have increased patient survival, such as PARP inhibitors [2] and immunotherapy [3], whereas traditional chemotherapy remains the primary systematic treatment for metastatic TNBC. Anthracyclines and taxanes, which are the backbone drugs of breast cancer treatment, have been used in the adjuvant and neoadjuvant settings. Once recurrence or metastasis occurs, gemcitabine alone or gemcitabine-based combination chemotherapy is one of the preferred treatment options for patients with TNBC who previously received anthracyclines and taxanes. Nevertheless, its use is limited by resistance in some tumor cells. The rate of primary gemcitabine resistance in patients with metastatic TNBC has been reported to reach 22–25% [4]. Even initially responsive patients eventually develop progressive disease and acquire secondary drug resistance. Thus, understanding the mechanisms of gemcitabine resistance and identifying new biomarkers remain major challenges.

Exosomes are nano-sized, membrane-bound vesicles that are released by various living cells [5]. Recent studies have suggested the hypothesis in which cancer exosomes are responsible for the drug resistance [6] and play a decisive role in the progression of multiple cancers [7, 8]. Primary drug-resistant tumors release exosomes that can modulate the biology of distant tumor cells and enhance their chemoresistance. Despite our growing understanding of the importance of and complexity of cancer exosomes and chemoresistance, the mechanisms of their regulation remain unclear.

Annexin A6 (ANXA6) belongs to the highly conserved annexin protein family. This protein can bind to acidic phospholipids in a calcium-dependent manner. ANXA6 controls membrane trafficking and cell signaling [9], thus interacting with cellular membranes in a dynamic, reversible, and regulated manner. Previous studies have reported the involvement of ANXA6 in both the positive and negative regulation of breast cancer cell growth, proliferation, and invasion [10]. The annexin family is correlated with drug resistance in various cancers, such as pancreatic [11] and ovarian cancers [12]. ANXA1 has been reported to be associated with resistance to tamoxifen in estrogen receptor-positive recurrent breast cancer [13] and trastuzumab resistance in HER-2 positive breast cancer [14]. However, no reported studies focused on the relationship between exosomal annexin expression and drug resistance.

In this study, we investigated whether exosomes derived from gemcitabine-resistant cells could mediate gemcitabine resistance in gemcitabine-sensitive cells, assessed the potential molecular mechanisms, and examined exosome protein levels in patient serum as a predictive biomarker for therapeutic responsiveness.

## Materials and methods

### Cell lines and reagents

MDA-MB-231 (MDA-231) cells were purchased from the American Type Culture Collection (USA). MDA-MB-231-HM (MDA-231-HM) cells with a high potential to metastasize to the lungs were established by our institute according to a previously described method [15]. The MDA-231 gemcitabine-resistant cell subline (MDA-231-R) and MDA-231-HM gemcitabine-resistant cell subline (MDA-231-HM-R) were established according to a previous study [16] by stepwise selection with increasing concentrations of gemcitabine (Eli Lilly, USA) with 12 cycles at a range of 12–720 nmol/l in the culture medium. All of the cell lines were tested and authenticated shortly before use. Breast cancer cell lines were cultured in the American Type Culture Collection-recommended media. Cells were cultured as a monolayer in 100% air with no CO_2_ in a humidified incubator at 37 °C and collected during their exponential growth phase. Cells were cultured for 24 h till attachment before experimental use. All cell lines were recently authenticated by STR profiling and tested for mycoplasma contamination. GW4869 was purchased from MedChemExpress (China). Lapatinib was purchased from Absin Bioscience Inc.,(China).

### Extraction, identification, and quantification of exosomes

Exosomes were isolated from cells using ExoQuick-TC for tissue Culture Media and Urine (SBI System Biosciences, Inc., USA) according to the manufacturer’s instructions. Briefly, 1 ml of ExoQuick reagent was added to 5 ml of cell culture supernatant, incubated for 12 h at 4 °C, and centrifuged at 1500×*g* for 30 min to obtain pelleted exosomes. The resulting exosomal preparation was dissolved in either PBS or RIPA buffer (Sigma-Aldrich, USA), depending on the application, and was either used immediately or stored at −80 °C. The exosomes were further used for the detection of marker proteins such as CD63 (1:1000, 25682-1-AP, Proteintech, USA), CD9 (1:1000, 20597-1-AP, Proteintech), and CD81 (1:1000, ab205606, Abcam, USA). The size and concentration of exosomes were determined via NanoSight tracking analysis using ZetaView PMX 110 (Particle Metrix, Germany). Exosomal preparations were analyzed for protein content using a BCA protein assay reagent kit (ThermoFisher Scientific, USA).

### Transmission electron microscopy of purified exosomes

In total, 10 µl of exosomes were applied to copper transmission electron microscopy grids (3.05 mm; 200 mesh) for 5 min, washed with PBS, and stained with 2% uranyl acetate for 3 min. Images were obtained using a transmission electron microscope (Tecnai G2 Spirit Biotwin, FEI Company, USA).

### PKH67 staining

Exosomes were labeled with the green fluorescent dye PKH67 (Sigma-Aldrich) per the manufacturer’s instructions. Briefly, the exosomes were diluted and suspended in 1 ml of diluent C. Four microliters of a PKH67 ethanolic dye solution were added to 1 ml of diluent C. Then, the PKH67 and exosome diluent solutions were mixed for 5 min at room temperature and washed three times with PBS. MDA-231 cells (3 × 10^5^ cells/well) were cultured on coverslips in 24-well plates containing 20 µg/ml PHK67-stained exosomes from different cells for 24 h, and the coverslips were gently washed with PBS and fixed with 4% paraformaldehyde solution. For fluorescence microscopy, nuclear staining was performed using DAPI (Sigma-Aldrich) and the early endosomes staining was performed using EEA1 (1:100, ab50313, Abcam, USA). Finally, the coverslips were placed on slides for viewing under a fluorescence microscope (Carl Zeiss Meditec AG, Germany) operated using Axiovision software (Carl Zeiss Meditec AG).

### Methyl-β-cyclodextrin treatment

Methyl-β-cyclodextrin (C4555-1G, Sigma-Aldrich) was dissolved in PBS. Cells were incubated with the solution at 37 °C for 4 h, rinsed extensively with L-15 using Vivaspin concentrators, and filter-sterilized before being assessed exosomal marker expressions using western blotting.

### Cell proliferation analysis

Cells were seeded into 96-well tissue culture plates at a density of 5 × 10^3^ cells/ml in 100 μl of culture medium and treated under various conditions for different periods of time. In addition, 10 μl of cell counting kit (CCK)-8 reagent (Dojindo Molecular Technologies Inc., Japan) were added to each well, and plates were incubated at 37 °C for 4 h. The viability of cancer cells was determined using the CCK-8 assay, and the optical density was measured at 450 nm using a plate reader (BioTek Company, USA). The inhibition ratios and the half-maximal inhibitory concentration (IC_50_) for each treatment condition were calculated using the optical density by GraphPad Prism® 7.0a software. The potency of cell proliferation inhibition was expressed as IC_50_.

### Colony formation

Cells were seeded into 6-cm dishes at a density of 1 × 10^3^ cells/ml and incubated with 15 nM gemcitabine and 20 μg/ml exosomes at 37 °C for 14 days. The dishes were then washed and fixed in 4% paraformaldehyde (Sigma-Aldrich, USA) for 15 min, and the cells were stained with crystal violet (Beyotime Biotechnology, China) at room temperature for 30 min. The morphology of cell colonies was recorded via photo imaging, and the cell colonies were quantified.

### Apoptosis analysis

The cells were seeded in 6-cm dishes with an initial density of 2 × 10^5^ cells/ml and treated with 20 μg/ml exosomes and 15 nM gemcitabine for 24 h. Then, cells were harvested and suspended using 195 μl of binding buffer. Next, they were stained with 5 μl of annexin V-FITC (Beyotime Biotechnology, China) in the dark for 5 min following staining with 10 μl of PI (Beyotime Biotechnology, China) at room temperature for 15 min. Finally, cells were examined using an FC500 flow cytometer (Beckman, USA). The data were analyzed using ModFit software (Verity Software House Inc., USA).

### Trypsin digestion and isobaric peptide labeling mass spectrometry (MS)

Proteins were reduced in 10 mM DTT at 37 °C for 1 h. Protein samples were then allowed to cool to room temperature, and cysteines were blocked via exposure to 30 mM IAA at 37 °C for 30 min in the dark. The extracted protein was then mixed according to groups at equal amounts and then precipitated with acetone overnight. After re-suspending the protein in 1 M urea buffer, the sample protein was digested with trypsin overnight. Subsequently, peptides were isotopically labeled with isobaric peptide labeling reagents (Applied Biosystems, Foster City, CA, USA) at room temperature for 2 h. The labeling reaction was then stopped by the addition of water. Next, the samples were separated and identified using a Triple TOF 4600 MS system (AB SCIEX, USA).

### Construction of ANXA6-knockdown and ANXA6-overexpressing TNBC cell lines

ShRNAs targeting the coding sequence of ANXA6 in pGIPZ lentiviral vectors, a nonsilencing shRNA control, or the empty vector were purchased from Genomeditech (Genomeditech, Shanghai, China) and shRNA sequences target ANXA6: 5′-TTCAGCATTGGTCCGAGTG-3′.

For experiments involving the overexpression of ANXA6, the PDS023_pL/IRES/GFP-Annexin A6 lentiviral vector and PCMV-C-Flag-Annexin A6 plasmid were subcloned. For PDS023_pL/IRES/GFP-Annexin A6, ANXA6 full-length was synthesized in Sangon (Sangon, Shanghai, China) and subcloned into PDS023_pL/IRES/GFP (as a vector) via BSMB1/ASCI. For PCMV-C-Flag-Annexin A6, ANXA6 were amplified by PCR using PDS023_pL/IRES/GFP-Annexin A6 clone as template and forward, 5′-CCGGAATTCATGGCCAAACCAGCACAGGGT-3′, and reverse, 5′-CCGCTCGAGCTAGTCCTCACCACCACAGAG-3′ primers, and then subcloned into pCMV-Flag (as a vector) via EcoRI/Xho1 sites.

The TNBC cells were transfected with shRNA, PDS023_pL/IRES/GFP-Annexin A6 lentiviral or PCMV-C-Flag-Annexin A6 plasmid using Lipofectamine 2000 transfection reagent according to the manufacturer’s protocol. Cells that stably express pGIPZ lentiviral shRNAs were selected with 1 µg/ml puromycin, cells that were infected with PDS023_pL/IRES/GFP-Annexin A6 were selected by 10 µg/ml Blasticidin and cells that stably express PCMV-C-Flag-Annexin A6 were selected by 0.7 mg/ml G418.

### RNA isolation, reverse-transcription, and qPCR

Total RNA was extracted from cells using a TRIzol kit (ThermoFisher Scientific, Waltham, MA, USA). A PrimeScript RT reagent kit (Takara, Kyoto, Japan) was used to synthesize complementary DNA. Next, qRT-PCR was performed to assess the mRNA expression of ANXA6 using 2 × SYBR Green real-time PCR Master Mix (Takara) and an Applied Biosystems 7300 Real Time PCR system (Applied Biosystems). The 2^−ΔΔCt^ method was used to quantify the data, and β-actin was used as a reference gene in the analysis. The forward primer was 5′-ACGGTTGATTGTGGGCCTG-3′, and the reverse primer was 5′-GTGCATCTGCTCATTGGTCC-3′.

### MS

MDA-231 cells pre-treated with 20 µg/ml exosomes for 24 h were lysed using SDT buffer. Next, 40 μl of trypsin buffer were added to each sample, and digestion was performed at 37 °C overnight. Then, the samples were re-suspended, separated using Easy nLC, and analyzed via online electrospray MS/MS. The experiments were performed on a Nano ACQUITY UPLC system (Waters Corporation, USA) connected to a Q-Exactive mass spectrometer (ThermoFisher Scientific, USA). Each sample was detected by liquid chromatography–MS/MS for 1 h, and the data were analyzed using PEAKS® Studio (ThermoFisher Scientific, USA).

### Immunoprecipitation assay

The cells were lysed by RIPA buffer for 15 min and centrifuged at 3000 rpm for 3 min at 4 °C. The supernatants were incubated overnight with 6 μl of anti-ANXA6, (12542-1-AP, 1:50, Proteintech, USA), anti-EGFR (ab52894, 1:20, Abcam, USA), anti-ubiquitin (ab19247, 1:100, Abcam, USA), or anti-Flag antibody (ab205606, 1:30, Abcam, USA) at 4 °C. The next day, samples were mixed with 40 μl of protein A agarose beads at 4 °C overnight that were pre-washed with RIPA buffer. Immunoprecipitation solutions were centrifuged at 3000 rpm for 3 min at 4 °C to collect the beads. Then, the beads were washed twice with cell lysis buffer. The complexes were eluted with 30 μl of SDS loading buffer, heated at 100 °C for 10 min, and subsequently analyzed via western blotting.

### GST pulldown assays

GST-tagged Annexin A6 in pGEX-4T-1 vector was used for bacterial expression in BL21 *E. coli* strain. GST fusion protein was isolated using glutathione resin (Clontech, USA) [17] and stored as 50% glycerol slurry. pCDNA(−)-HA-EGFR transfected HEK293T cells were washed with PBS and incubated on ice for 15 min with lysis buffer (50 mM Tris-HCl, pH 7.5, 150 mM NaCl, 1.5 mM MgCl_2_, 1 mM EDTA, 1% Triton X-100, and 10% glycerol). Lysate was clarified by centrifugation and incubated with glutathione resin loaded with GST-Annexin A6 (2 h, 4 °C). The resin was then collected by centrifugation and washed three times with lysis buffer, and the amount of HA-EGFR bound to Annexin A6 beads was detected by western blot analysis.

### Ubiquitination assay

Briefly, MDA-231 and transfected MDA-231-A6 cells were plated in six-well plates (4 × 10^5^ cells/well) and cultured at 37 °C in an incubator for 24 h. Next, the cells were treated with 15 μg/ml cycloheximide (Sigma-Aldrich) for 0, 1, 2, or 4 h or 20 μM MG132 (Sigma-Aldrich) or 10 mmol/l 3-methyladenine (Sigma-Aldrich) for 24 h and collected. Immediately, the cells were lysed using RIPA lysis buffer on ice for 20 min, centrifuged at 10,000 rpm for 15 min at 4 °C, and subsequently analyzed via western blotting.

### Western blotting

Western blot analysis was performed according to a previously described previously [18]. The following antibodies were used: anti-ANXA6 (1:500, 12542-1-AP, Proteintech, USA), anti-ANXA1 (1:1000, 21990-1-AP, Proteintech, USA), anti-EGFR (1:1000, ab52894, Abcam, USA), anti-TFPI (1:2000, 66842-1-Ig, Proteintech, USA), anti-COL8A1 (1 µg/ml, ab100988, Abcam, USA), anti-HLA-B (1:1000, 17260-1-AP, Proteintech, USA), anti-Flag (ab205606, 1:1000, Abcam, USA) and anti-fibronectin (ab2413, 1:3000, Abcam, USA). The signals were visualized using a luminescent image analyzer (ImageQuant LAS4000 mini, USA). GAPDH (1:10,000, #5174, Cell Signaling Technology, USA) and β-actin (1:1000, ab8226, Abcam, USA) was used as loading controls.

### Immunohistochemistry and scoring

Using an institution review board-approved sample collection protocol at the Fudan University Shanghai Cancer Center, subjects consented to tissue collections. A total of 81 patients, who received curative resection for triple-negative breast cancer at authors’ institutes from August 2015 to August 2016, were enrolled. Hematoxylin and eosin (H&E)-stained slides from 81 patients with TNBC were reviewed and identified by two experienced pathologists. The representative cores were pre-marked in the paraffin blocks, and tissue cylinders with a diameter of 1.0 mm were punched from the marked areas, and then incorporated into a recipient paraffin block. Sections with a thickness of 4 μm were placed on slides coated with 3-aminopropyltriethoxysilane and dried at 65 °C for 2 h. The slides were incubated with the primary antibodies against ANXA6 (1:100, 12542-1-AP, Proteintech, USA) and EGFR (1:100, ab52894, Abcam, USA) overnight at 4 °C in a moist chamber, and then conjugated with secondary antibody labeled with horseradish peroxidase (K500711-2, EnVision™ Detection Systems Peroxidase/DAB, Rabbit/Mouse, DAKO, Denmark) for 40 min at room temperature, and stained with DAB according to the manufacturer’s instructions. The slides were then counterstained with hematoxylin, differentiated using hydrochloric alcohol, dehydrated in an ascending ethanol series, cleared in xylene, sealed with neutral balsam, and lastly, scanned by Aperio Image Scope (Leica Biosystems Imaging, Inc., Germany). The data were analyzed using Image-Pro Plus Analysis software. The integrated optical density was calculated for each slide.

### Exosome isolation from patient serum

Using an institution review board-approved sample collection protocol at the Fudan University Shanghai Cancer Center, subjects consented to venous blood collection. The study complied with all relevant ethical regulations regarding research involving human participants. A total of 21 patients, who were diagnosed with metastatic TNBC and treated with gemcitabine-based chemotherapy as first line at authors’ institutes from October 2017 to November 2018, were enrolled. The immunohistochemical subtype and lymph node status of each patient are reported in Supplementary Table 2. Four milliliters of blood were collected into tubes for all subjects before the initiation of first-line gemcitabine-based chemotherapy. We purified exosomes from 500 μl of patient serum using ExoQuick exosome precipitation solution (SBI System Biosciences, Inc., USA) according to the manufacturer’s protocol.

### ELISA

The levels of exosomal ANXA6 from patient serum were detected using a human Annexin A6 ELISA kit (Shanghai Enzyme-linked Biotechnology Co., Ltd, China) following the manufacturer’s instructions.

### Statistical analysis

The results are presented as the mean ± SD for three independent experiments. Experiments that involved two experimental groups were analyzed using a two-sided Student’s *t*-test or analysis of one-way variance with 95% confidence intervals. The variance similar between the groups that are being statistically compared. *P* < 0.05 indicated statistical significance. All statistical analyses were performed using the GraphPad Prism® 7.0a software and the Statistical Package for Social Sciences Version 22.0 (SPSS 16.0, USA).

## Results

### Establishment of resistant cell lines

MDA-231-R and MDA-231-HM-R cells were established as previously described [16]. The IC_50_ values in MDA-231, MDA-231-R, MDA-231-HM, and MDA-231-HM-R cells were 8.86, 152.70, 11.62, and 87.46 nM, respectively (Supplementary Fig. 1). The results illustrated that MDA-231-R cells were 17.2-fold more resistant to the drug than MDA-231 cells. MDA-231-HM-R cells were 7.5-fold more resistant to the drug than MDA-231-HM cells, indicating the successful establishment of resistant cell sublines.

### TNBC cell-derived exosomes and their internalization

To determine the participation of tumor-derived exosomes in chemoresistance, exosomes were isolated from the supernatants of TNBC cells, including those derived from MDA-231 (231-S-exo), MDA-231-HM (231-HM-S-exo), MDA-231-R (231-R-exo), and MDA-231-HM-R cells (231-HM-R-exo). Exosomes were round in shape with diameter of 130–150 nm (Supplementary Fig. 2A, B) as examined using transmission electron microscopy and nanoparticle tracking analysis. Exosomes were enriched in the exosomal markers CD9, CD63, and CD81, and were almost absent in fibronectin, which was more associated with non-vesicular fractions [19] (Supplementary Fig. 2C, D). PKH67-labeled exosomes were internalized and partially colocalized with the early endosomes in the cytoplasm of cells (Supplementary Fig. 2E–H), indicating that exosomes could be taken up and internalized by sensitive cells. The differences in the quantity and distribution of these internalized exosomes and their colocalization with the early endosomes between the two groups were not analyzed. Next, we altered exosomal integrity using methyl β-cyclodextrin, which resulted in the loss of CD9, CD63, and CD81 expression (Supplementary Fig. 3). The results above confirmed the successful extraction of exosomes.

### Gemcitabine resistance transfer by exosomes

**Fig. 1:**
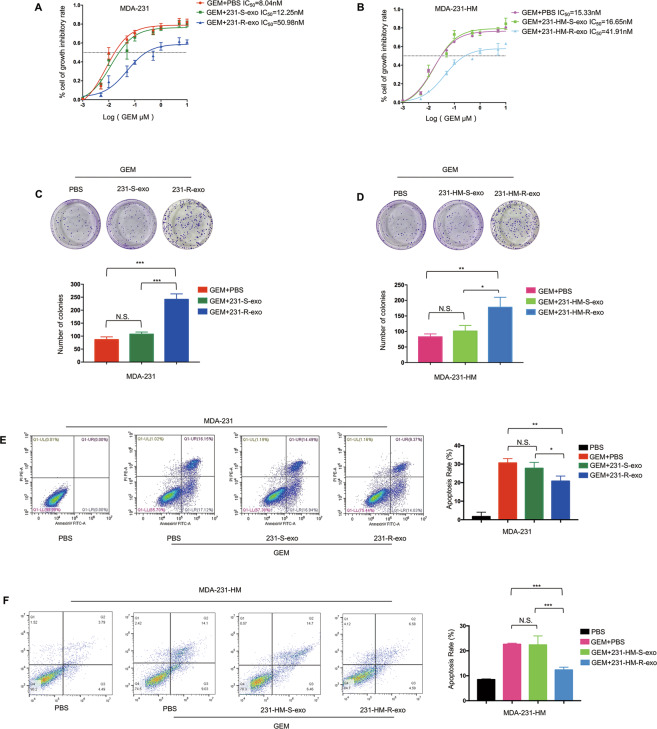
Exosomes derived from resistant cells enhance gemcitabine (GEM) resistance in triple-negative breast cancer. **A**, **B** IC_50_ values determined using the CCK-8 assay in MDA-231 and MDA-231-HM cells treated with GEM + PBS, GEM + sensitive cell-derived exosomes (231-S-exo or 231-HM-S-exo), and GEM + resistant cell-derived exosomes (231-R-exo or 231-HM-R-exo) for 48 h. All experiments were repeated three times, and the representative results are presented. **C**, **D** Colony formation analysis in MDA-231 and MDA-231-HM cells treated with GEM + PBS, GEM + sensitive cell-derived exosomes (231-S-exo or 231-HM-S-exo), or GEM + resistant cell-derived exosomes (231-R-exo or 231-HM-R-exo) for 24 h and incubated for 7 days. **E**, **F** Flow cytometric analyses of apoptotic cells in MDA-231 and MDA-231-HM cells treated with PBS, GEM + PBS, GEM + sensitive cell-derived exosomes (231-S-exo or 231-HM-S-exo), or GEM + resistant cell-derived exosomes (231-R-exo or 231-HM-R-exo) for 24 h. Quantitative data are presented as the mean ± SD of triplicate experiments. *P < 0.05, **P < 0.01, ***P < 0.001, and ****P < 0.0001.

To determine whether resistant cell-derived exosomes could induce gemcitabine resistance in sensitive cells, cell proliferation, colony formation, and apoptosis analyses were performed. MDA-231 cells were treated with PBS, 20 µg/ml 231-S-exo or 20 µg/ml 231-R-exo and gemcitabine at different concentrations (0.001, 0.005, 0.01, 0.05, 0.1, 0.5, 1, 5, 10 µM) for 48 h. As shown in Fig. 1A, MDA-231 cells became comparatively less responsive to gemcitabine after exosomal treatment, resulting in a higher IC_50_. The same result was obtained in MDA-231-HM cells (Fig. 1B). In the cell colony analysis, MDA-231 cells were treated with PBS, 20 µg/ml 231-S-exo or 20 µg/ml 231-R-exo and 15 nM gemcitabine for 14 days. MDA-231-HM cells were treated with PBS, 20 µg/ml 231-HM-S-exo, or 20 µg/ml 231-HM-R-exo and 15 nM gemcitabine for 14 days. The number of colonies was significantly larger in the gemcitabine + 231-R-exo group (Fig. 1C, gemcitabine + PBS vs. gemcitabine + 231-R-exo, *P* = 0.0004) and gemcitabine + 231-HM-R-exo groups (Fig. 1D, gemcitabine + PBS vs. gemcitabine + 231-HM-R-exo, *P* = 0.0083).

In Fig. 1E, F, gemcitabine-induced apoptosis was assessed in the presence of cell-derived exosomes. MDA-231 cells were treated with PBS, 15 nM gemcitabine and PBS, 15 nM gemcitabine and 20 µg/ml 231-S-exo, or 15 nM gemcitabine and 20 µg/ml 231-R-exo for 24 h. MDA-231-HM cells were treated with PBS, 15 nM gemcitabine and PBS, 15 nM gemcitabine and 20 µg/ml 231-HM-S-exo, or 15 nM gemcitabine and 20 µg/ml 231-HM-R-exo for 24 h. The percentages of apoptotic cells in the PBS, gemcitabine, gemcitabine and sensitive cells-derived exosomes, and gemcitabine and resistant cell-derived exosome groups were 1.71 ± 1.38%, 30.66 ± 1.32%, 27.79 ± 1.82%, and 20.83 ± 1.59% for MDA-231 cells and 8.50 ± 0.13%, 22.64 ± 0.24%, 22.39 ± 2.07%, and 12.35 ± 0.64% for MDA-231-HM cells. The rates of apoptosis were significantly lower in the gemcitabine + 231-R-exo group than in the gemcitabine + PBS group (*P* = 0.0089, Fig. 1E) and the gemcitabine + 231-S-exo group (*P* = 0.045, Fig. 1E). Concerning MDA-231-HM cells, a lower apoptosis rate was also observed in the gemcitabine + 231-HM-R-exo group than in the gemcitabine + PBS (*P* < 0.001, Fig. 1F) and gemcitabine + 231-HM-S-exo groups (*P* < 0.001, Fig. 1F). These results indicated that the resistant cell-derived exosomes inhibited the chemotherapeutic effects of gemcitabine.

To further explore whether exosomes played an important role in this effect, we suppressed exosome production using GW4869, a pharmacological inhibitor of neutral sphingomyelinase-2. As shown in Supplementary Fig. 4A, the number of exosomes was reduced dramatically after GW4869 treatment, but GW4869 had no influence on cell viability and intracellular ANXA6 levels (Supplementary Fig. 4B, C). The culture medium from GW4869-treated MDA-231-R cells failed to induce gemcitabine resistance in sensitive cells (Supplementary Fig. 4D), indicating the critical role of exosomes for the transfer of resistance.

### Exosomes affect gemcitabine resistance via exosomal ANXA6 upregulation

**Fig. 2:**
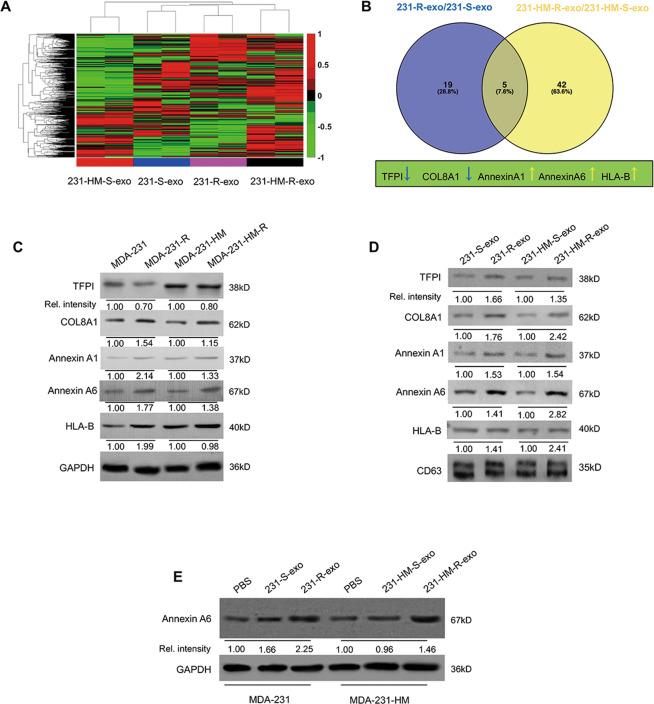
Annexin A6 (ANXA6) is upregulated in gemcitabine-resistant cell-derived exosomes. **A** The heatmap presents differentially expressed proteins between two pairs of exosomes (231-S-exo and 231-R-exo, 231-HM-S-exo and 231-HM-R-exo). **B** The Venn diagram of overlap between 231-R-exo/231-S-exo and 231-HM-R-exo/231-HM-S-exo. **C**, **D** TFPI, COL8A1, annexin A1, ANXA6 and HLA-B protein levels in MDA-231, MDA-231-R, MDA-231-HM, and MDA-231-HM-R cells and in 231-S-exo, 231-R-exo, 231-HM-S-exo, and 231-HM-R-exo. **E** The changes of ANXA6 in MDA-231 and MDA-231-HM cells treated with PBS, sensitive cell-derived exosomes (231-S-exo or 231-HM-S-exo), or resistant cell-derived exosomes (231-R-exo or 231-HM-R-exo). All the experiments were repeated three times, and the representative results are presented.

Isobaric peptide labeling—liquid chromatography–MS/MS was performed to assess differential protein expression between two pairs of exosomes (231-S-exo and 231-R-exo, 231-HM-S-exo and 231-HM-R-exo). The heatmap presented in Fig. 2A revealed substantial differences. Five proteins with significant changes were detected in both pairs of exosomes (*P* < 0.05). Underexpressed proteins in both 231-R-exo and 231-HM-R-exo included TFP-1 and COL8A1, whereas overexpressed proteins included ANXA1, ANXA6, and HLA-B (Fig. 2B). As presented in Fig. 2C, D, the overexpression of COL8A1, ANXA1, and ANXA6 were in line with the liquid chromatography–MS/MS results in cells and exosomes, and ANXA6 was the most prominently upregulated protein. In a previous study, the Key Laboratory of Breast Cancer in our hospital explored the gemcitabine resistance mechanism, and its microarray data (deposited in the National Center for Biotechnology Information Gene Expression Omnibus database under the accession number GSE63140) revealed an increase of ANXA6 expression but no changes in ANXA1 expression [16]. As presented in Fig. 2E, when MDA-231 and MDA-231-HM cells were treated with PBS, sensitive cell-derived exosomes (231-S-exo or 231-HM-S-exo), or resistant cell-derived exosomes (231-R-exo and 231-HM-R-exo) for 24 h, ANXA6 was upregulated in the resistant cell-derived exosomes treated group. Therefore, ANXA6 may be the key protein involved in gemcitabine resistance mechanisms.

**Fig. 3:**
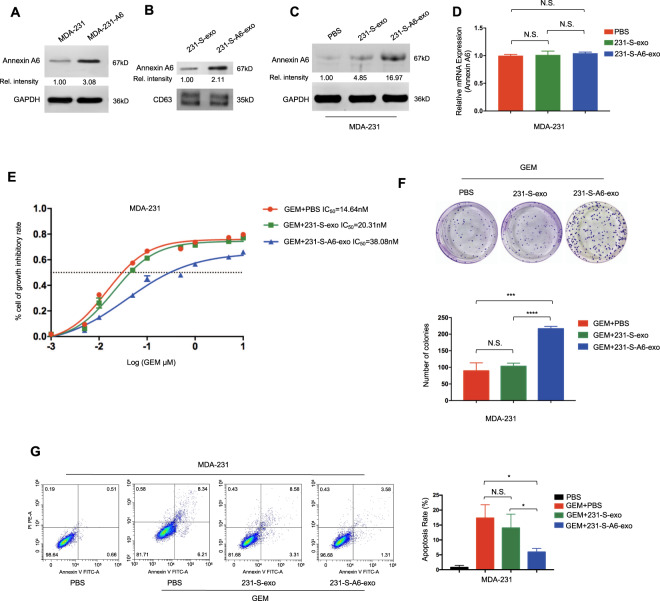
Chemoresistant cell-derived exosomes enhance gemcitabine (GEM) resistance via exosomal annexin A6 (ANXA6) upregulation. **A** The Annexin A6 (ANXA6) was overexpressed in the ANXA6 stably overexpressing MDA-231 cells (MDA-231-A6). **B** ANXA6 was overexpressed in MDA-231-A6 cell-derived exosomes (231-S-A6-exo). **C** MDA-231 cells were pre-treated with PBS, 231-S-exo, or 231-S-A6-exo for 24 h and then subjected to western blotting. **D** qRT-PCR analysis of ANXA6 levels in MDA-231 cells treated with PBS, 231-S-exo, or 231-S-A6-exo for 24 h. **E** IC_50_ values determined using the CCK-8 assay in MDA-231 cells treated with GEM + PBS, GEM + 231-S-exo, or GEM + 231-S-A6-exo for 48 h. **F** Colony formation in MDA-231 cells treated with GEM + PBS, GEM + 231-S-exo, or GEM + 231-S-A6-exo for 24 h and incubated for 7 days. **G** Flow cytometric analyses of apoptosis in MDA-231 cells exposed to PBS, GEM + PBS, GEM + 231-S-exo, or GEM + 231-S-A6-exo for 24 h. All the experiments were repeated three times, and the representative ones are presented. Quantitative data are presented as the mean ± SD of triplicate experiments. *P < 0.05, **P < 0.01, ***P < 0.001, and ****P < 0.0001.

To explore the possible mechanisms of ANXA6-involved gemcitabine resistance, ANXA6-overexpressing MDA-231 cells (MDA-231-A6) were established (Fig. 3A), and ANXA6 was also overexpressed in MDA-231-A6 cells-derived exosomes (231-S-A6-exo) (Fig. 3B), which was in line with the cellular expression data. ANXA6 was upregulated when MDA-231 cells were treated with 231-S-A6-exo (Fig. 3C), which had no influence on intracellular ANXA6 mRNA levels (Fig. 3D). Compared with the effects of 231-S-exo, 231-S-A6-exo conferred resistance potential. MDA-231 cells were treated with PBS, 20 µg/ml 231-S-exo, or 20 µg/ml 231-S-A6-exo and gemcitabine at different concentrations (0.001, 0.005, 0.01, 0.05, 0.1, 0.5, 1, 5, and 10 µM) for 48 h. MDA-231 cells became less responsive to gemcitabine after 231-S-A6-exo treatment, resulting in a higher IC_50_ (Fig. 3E). In the cell colony analysis, MDA-231 cells were treated with PBS, 20 µg/ml 231-S-exo, or 20 µg/ml 231-S-A6-exo and 15 nM gemcitabine for 24 h. The number of colonies was significantly larger in the gemcitabine + 231-S-A6-exo group (Fig. 3F, gemcitabine + PBS vs. gemcitabine + 231-S-A6-exo, *P* = 0.0009). Gemcitabine-induced apoptosis in the presence of cell-derived exosomes was assessed, as presented in Fig. 3G. MDA-231 cells were treated with PBS, 15 nM gemcitabine and PBS, 15 nM gemcitabine and 20 µg/ml 231-S-exo, or 15 nM gemcitabine and 20 µg/ml 231-S-A6-exo for 24 h. The percentages of apoptotic cells in the four groups were 0.78 ± 0.39%, 17.36 ± 2.55%, 14.04 ± 2.64% and 5.94 ± 0.69%, respectively. The apoptotic rates were significantly lower in the gemcitabine + 231-S-A6-exo group than in the gemcitabine + PBS and gemcitabine + 231-S-exo groups (*P* = 0.0124 and *P* = 0.0412, respectively).

**Fig. 4:**
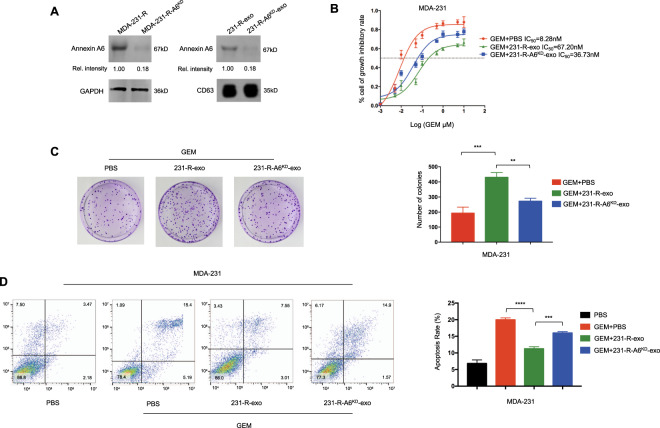
Knockdown Annexin A6 (ANXA6) rescues exosome-induced gemcitabine resistance. **A** ANXA6 expression was reduced in ANXA6-knockdown MDA-231-R cells (MDA-231-R-A6^KD^) and their exosomes (231-R-A6^KD^-exo). **B** IC_50_ values determined using the CCK-8 assay in MDA-231 cells treated with GEM + PBS, GEM + 231-R-exo, or GEM + 231-R-A6^KD^-exo for 48 h. **C** Colony formation in MDA-231 cells treated with GEM + PBS, GEM + 231-R-exo, or GEM + 231-R-A6^KD^-exo for 24 h and incubated for 7 days. **D** Flow cytometric analyses of apoptosis in MDA-231 cells treated with PBS, GEM + PBS, GEM + 231-R-exo, or GEM + 231-R-A6^KD^-exo for 24 h. All experiments were repeated three times, and the representative results are presented. Quantitative data are presented as the mean ± SD of triplicate experiments. *P* < 0.05, **P < 0.01, ***P < 0.001, and ****P < 0.0001.

ANXA6-knockdown MDA-231-R cells (MDA-231-R-A6^KD^) were also established (Fig. 4A). The addition of MDA-231-R-A6^KD^-derived exosomes (231-R-A6^KD^-exo) inhibited the proliferation of MDA-231 cells. MDA-231 cells became more responsive to gemcitabine after 231-R-A6^KD^-exo treatment (Fig. 4B). In the cell colony analysis, the number of colonies was smaller in the gemcitabine + 231-R-A6^KD^-exo group (Fig. 4C, gemcitabine + 231-R-exo vs. gemcitabine + 231-S-A6-exo, *P* = 0.0054). As presented in Fig. 4D, the apoptosis rate was higher in the gemcitabine + 231-R-A6^KD^-exo group than in gemcitabine + 231-R-exo groups (*P* = 0.002). These results indicated that exosomes affect gemcitabine resistance via exosomal ANXA6 upregulation.

### The interaction of ANXA6 and EGFR induces gemcitabine resistance by inhibition of EGFR ubiquitination and degradation

**Fig. 5:**
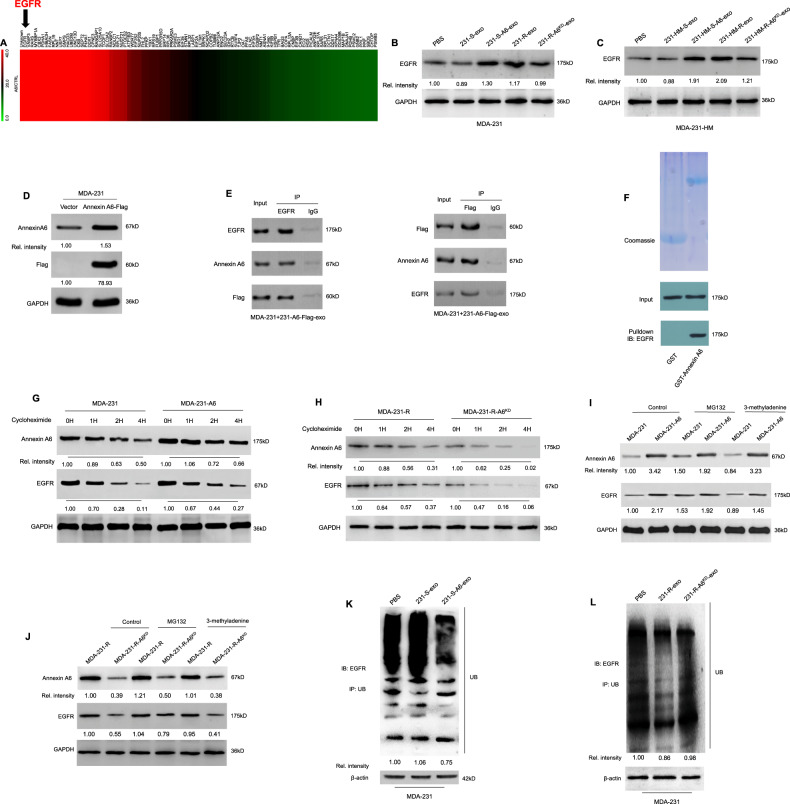
The interaction of ANXA6 and EGFR inhibit EGFR ubiquitination and degradation. **A** Mass spectrographic analysis revealed the different gene expression patterns between MDA-231 cells treated with 231-S-exo and 231-S-A6-exo. The second most strongly upregulated gene was EGFR. **B** EGFR was upregulated in MDA-231 cells treated with 231-S-A6-exo and 231-R-exo. EGFR was downregulated in MDA-231 cells treated with 231-R-A6^KD^-exo when compared with MDA-231 cells treated with 231-R-exo. **C** EGFR was upregulated in MDA-231-HM cells treated with 231-HM-S-A6-exo and 231-HM-R-exo. EGFR was downregulated in MDA-231-HM cells treated with 231-HM-R-A6^KD^-exo when compared with MDA-231-HM cells treated with 231-HM-R-exo. **D** ANXA6-Flag was overexpressed in stably transfected MDA-231 cells (MDA-231-A6-Flag). **E** Co-immunoprecipitation (IP) was performed using an IgG antibody control or antibodies against ANXA6, Flag, or EGFR on lysates from MDA-231 cells treated with MDA-231-A6-Flag cell-derived exosomes (231-A6-Flag-exo). IP products with IgG and specific antibodies were resolved via western blotting and probed for ANXA6, Flag, and EGFR. **F** The expression of GST and GST-Annexin A6 were examined with Coomassie blue staining (top). GST pulldown assays with GST or GST-Annexin A6 purified from *E. coli* and in vitro translated EGFR. EGFR interacted with GST-Annexin A6 as detected by EGFR Western blot (bottom). **G**, **H** The expression levels of EGFR and ANXA6 were examined by western blotting in MDA-231, MDA-231-A6, MDA-231-R, and MDA-231-R-A6^KD^ cells treated with 15 µg/ml cycloheximide for 0, 1, 2, or 4 h. **I**, **J** The expression levels of ANXA6 and EGFR were examined by western blotting in MDA-231, MDA-231-A6, MDA-231-R, and MDA-231-R-A6^KD^ cells treated with the proteasome inhibitor MG132 (20 µM) and autophagy inhibitor 3-methyladenine (10 mmol/l) for 24 h. **K**, **L** Ubiquitination assay detected the inhibition of EGFR ubiquitination and degradation in MDA-231 cells treated with PBS, 231-S-exo and 231-S-A6-exo or PBS, 231-R-exo and 231-R-A6^KD^-exo. All experiments were repeated three times, and the representative results are presented.

To reveal the mechanisms of ANXA6-mediated gemcitabine resistance, MDA-231 cells were treated with 20 µg/ml 231-S-exo or 231-S-A6-exo for 24 h, followed by mass spectrographic analysis. The heatmap analysis revealed different gene expression patterns between the two groups. The most strongly upregulated protein was an unknown protein, followed by EGFR (Fig. 5A). EGFR upregulation was confirmed via western blotting when MDA-231 and MDA-231-HM cells were treated with sensitive cell-derived exosomes overexpressed with ANXA6 (231-S-A6-exo or 231-HM-S-A6-exo) or resistant cell-derived exosomes (231-R-exo or 231-HM-R-exo, Fig. 5B, C). ANXA6-Flag–overexpressing MDA-231 cells (MDA-231-A6-Flag) were established (Fig. 5D). The interactions between exosomal ANXA6 and EGFR were examined using Flag pulldown or EGFR pulldown assays in MDA-231 cells treated with MDA-231-A6-Flag cell-derived exosomes (231-A6-Flag-exo). Co-IP experiments demonstrated that exosomal ANXA6-Flag and EGFR interacted with each other in MDA-231 cells (Fig. 5E). The GST pulldown assay confirmed ANXA6–EGFR interactions (Fig. 5F). This indicated that exosomal ANXA6-Flag entered MDA-231 cells and interacted with EGFR.

To examine the role of ANXA6 in regulating EGFR protein stability, MDA-231, MDA-231-A6, MDA-231-R, and MDA-231-R-A6^KD^ cells were treated with 15 µg/ml cycloheximide for 0, 1, 2, or 4 h. Inhibition of de novo protein synthesis by cycloheximide decreased EGFR and ANXA6 protein levels. However, the extent of EGFR downregulation was smaller in MDA-231-A6 and MDA-231-R cells (Fig. 5G, H). These findings indicated that the degradation of EGFR was impaired by ANXA6 overexpression. Given the existence of two primary pathways of protein degradation, namely the ubiquitin-proteasome and lysosomal degradation pathways, the cells were treated with the proteasome inhibitor MG132 and the autophagic/lysosomal protein degradation inhibitor 3-methyladenine. Treatment with MG132 essentially upregulated EGFR expression in MDA-231 cells and MDA-231-R-A6^KD^ cells, whereas 3-methyladenine had no effect (Fig. 5I, J), which indicated that EGFR degradation, at least partially, was attributable to the ubiquitin-proteasomal pathway. As presented in Fig. 5K, the ubiquitination assay detected the inhibition of EGFR ubiquitination and degradation in MDA-231 cells treated with 231-S-A6-exo compared with the findings in MDA-231 cells treated with PBS and 231-S-exo. On the contrary, the inhibition of EGFR ubiquitination and degradation was reversed in MDA-231 cells treated with 231-R-A6^KD^-exo compared with the findings in MDA-231 cells treated with 231-R-exo (Fig. 5L). The aforementioned results indicated that exosomal ANXA6 was involved in the suppression of EGFR ubiquitination and degradation.

**Fig. 6:**
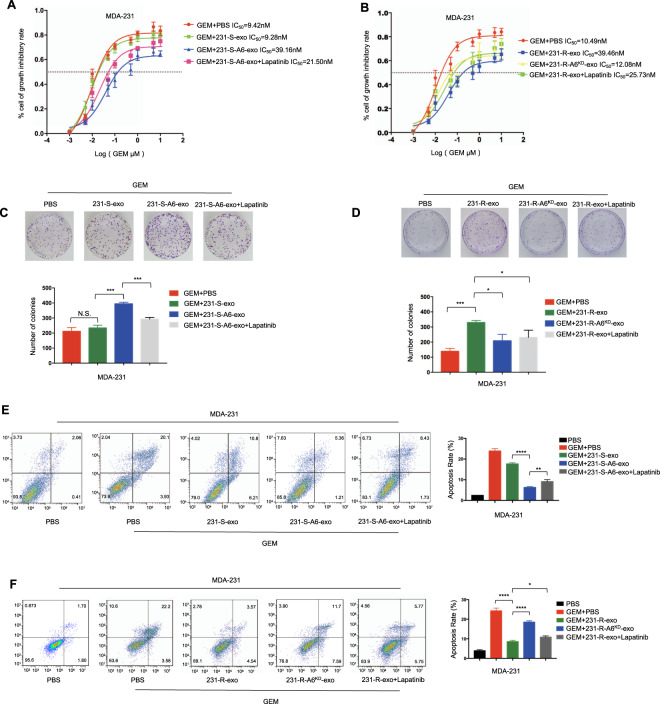
EGFR-TKI reverses gemcitabine (GEM) resistance induced by exosomal ANXA6. **A**, **B** IC_50_ values determined using the CCK-8 assay in MDA-231 cells treated with GEM + PBS, GEM + 231-S-exo, GEM + 231-S-A6-exo, and GEM + 231-S-A6-exo + lapatinib for 48 h or GEM + PBS, GEM + 231-R-exo, GEM + 231-R-A6^KD^-exo, and GEM + 231-R-exo + lapatinib for 48 h. **C**, **D** Colony formation in MDA-231 cells treated with GEM + PBS, GEM + 231-S-exo, GEM + 231-S-A6-exo, or GEM + 231-S-A6-exo + lapatinib for 24 h and incubated for 7 days or GEM + PBS, GEM + 231-R-exo, GEM + 231-R-A6^KD^-exo, or GEM + 231-R-exo + lapatinib for 24 h and incubated for 7 day. **E**, **F** Flow cytometric analyses of apoptosis in MDA-231 cells treated with PBS, GEM + PBS, GEM + 231-S-exo, GEM + 231-S-A6-exo, or GEM + 231-S-A6-exo + lapatinib for 24 h or PBS, GEM + PBS, GEM + 231-R-exo, GEM + 231-R-A6^KD^-exo, or GEM + 231-R-exo + lapatinib for 24 h. All experiments were repeated three times, and the representative results are presented. Quantitative data are presented as the mean ± SD of triplicate experiments. *P < 0.05, **P < 0.01, ***P < 0.001, and ****P < 0.0001.

To further explore whether EGFR was involved in gemcitabine resistance mediated by exosomal ANXA6, MDA-231-cells were treated with 15 nM gemcitabine and PBS, 20 µg/ml 231-S-exo, 20 µg/ml 231-S-A6-exo, or 20 µg/ml 231-S-A6-exo and 0.5 μM lapatinib or 15 nM gemcitabine and PBS, 20 µg/ml 231-R-exo, 20 µg/ml 231-R-A6^KD^-exo, or 20 µg/ml 231-R-exo and 0.5 μM lapatinib. The CCK-8 assay demonstrated that MDA-231 cells became more responsive to gemcitabine after exposure to lapatinib (Fig. 6A, B). The number of colonies was also smaller in the gemcitabine + 231-S-A6-exo + lapatinib group (Fig. 6C, gemcitabine + 231-S-A6-exo vs. gemcitabine + 231-S-A6-exo + lapatinib, *P* = 0.0004; Fig. 6D, gemcitabine + 231-R-exo vs. gemcitabine + 231-R-exo + lapatinib, *P* = 0.00315). Moreover, flow cytometry illustrated that the percentage of apoptotic cells was higher in the gemcitabine + 231-S-A6-exo + lapatinib group than in the gemcitabine + 231-S-A6-exo group (Fig. 6E, *P* = 0.0055) and gemcitabine + 231-R-exo + lapatinib group than in the gemcitabine + 231-R-exo group (Fig. 6F, *P* = 0.0430). These results indicated that gemcitabine resistance induced by exosomal ANXA6 could be reversed by lapatinib. Taken together, these results suggested that exosomal ANXA6 inhibited EGFR ubiquitination and degradation, thereby mediating gemcitabine resistance.

### Baseline exosomal ANXA6 in serum from patients with TNBC might be a potential predictor of responsiveness to first-line gemcitabine-based chemotherapy

**Fig. 7:**
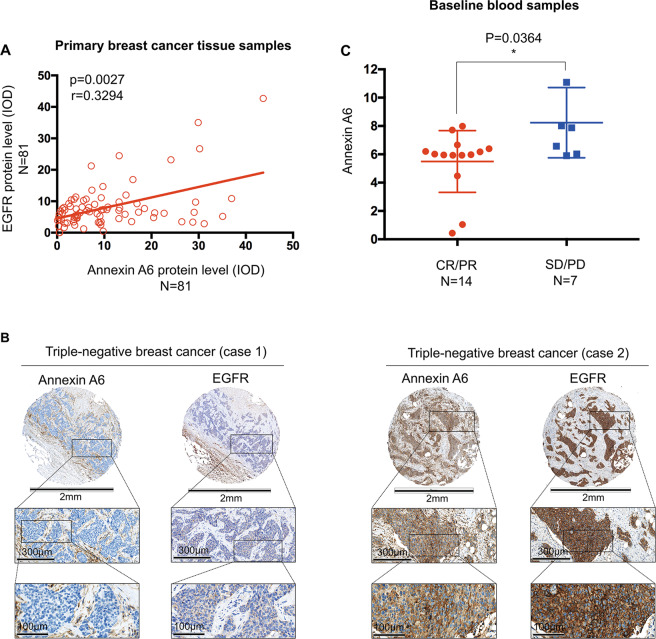
The positive correlations of ANXA6 and EGFR and exosomal ANXA6 are less enriched in gemcitabine-sensitive patients. **A** Scatter plot of ANXA6 and EGFR protein levels as assessed via immunohistochemistry using a tissue microarray constructed by samples from 81 patients with triple-negative breast cancer. The baseline characteristics of the 81 patients are shown in Supplementary Table 1. **B** Representative images of ANXA6 and EGFR immunohistochemistry in primary triple-negative breast cancer tissue. Low ANXA6 protein expression in a tumor with low EGFR protein expression (case 1) and high ANXA6 protein expression in a tumor with high EGFR protein expression (case 2). **C** ELISA revealed that the baseline levels of exosomal ANXA6 isolated from the serum of patients with triple-negative breast cancer were lower in sensitive patients with the best response of complete or partial responses (*n* = 14) than in those with stable or progressive disease (*n* = 7). The tumor response was assessed at every two or three cycles. The baseline characteristics of 21 patients are shown in Supplementary Table 2.

We investigated the protein expression of ANXA6 and EGFR in cancer tissue from 81 patients with TNBC. Their characteristics at baseline are shown in Supplementary Table 1. As shown in Fig. 7A, B, there was a positive correlation between ANXA6 and EGFR protein expression in primary TNBC tissues (r = 0.3294, *P* = 0.0027), i.e., low ANXA6 protein expression in tumors with low EGFR protein expression (case 1) and high ANXA6 protein expression in tumors with high EGFR protein expression (case 2). Finally, the exosomal ANXA6 expression levels were measured in 21 patients with TNBC, whose baseline characteristics are shown in Supplementary Table 2. Notably, ANXA6 levels in exosomes at baseline were lower in patients with a best overall response of partial or complete responses than in those with stable or progressive disease, (Fig. 7C, *P* = 0.0364), which strongly suggests that exosomal ANXA6 might be a potential predictor of gemcitabine-based chemotherapy responsiveness. Although the sample size was small, these data support the notion that high exosomal ANXA6 levels were related to gemcitabine-based chemotherapy resistance in patients with TNBC.

## Discussion

Chemotherapy is the backbone of systemic treatment for metastatic TNBC, and it improves survival by inhibiting cancer cell growth and invasion. However, primary or secondary drug resistance limits the benefits of chemotherapy treatments and thereafter impairs patient prognosis. Our results revealed that exosomal ANXA6 derived from gemcitabine-resistant cancer cells interacted with EGFR and induced gemcitabine resistance by inhibiting EGFR ubiquitination and degradation. These biological interactions between exosomal ANXA6 and EGFR in TNBC were mirrored by statistical evidence that exosomal ANXA6 levels in serum from patients with TNBC who received first-line gemcitabine-based chemotherapy were also predictive of therapeutic response.

Annexin proteins family are Ca^2+^-binding membrane-associated proteins that have a close relationship with cancers. They are associated with drug resistance, including ANXA2 in nasopharyngeal carcinoma [20], ANXA3 in colorectal [21] and ovarian cancers [22], and ANXA5 in lung cancer [23]. ANXA6 controls membrane trafficking and cell signaling [9]. Previous studies reported its involvement in both the positive and negative regulation of breast cancer cells [10]. ANXA6 is selectively enriched in cancer-originated exosomes [24, 25]. It was reported that chemotherapy-elicited exosomes are enriched in ANXA6 that facilitates the establishment of lung metastasis, and the patients with elevated ANXA6 levels have progressive disease in the neoadjuvant setting, which strongly suggests that chemoresistance is potentially related with exosome-associated ANXA6 and it was of cancer cell origin [26]. In fact, the potential of ANXA6 as a biomarker for cancer has been previously investigated. The detection of ANXA6 may be useful as a serum biomarker for esophageal adenocarcinoma [27] and pancreatic cancer [28]. Nevertheless, few studies demonstrated the association of ANXA6/exosomal ANXA6 and drug resistance in cancers. Our data provided evidence that the presence of ANXA6 in blood samples before gemcitabine-based chemotherapy may be a reliable predictor of tumor cell responsiveness to treatment.

EGFR is a member of the ErbB receptor family. Approximately 40% of patients with TNBC overexpress EGFR [29]. Our results revealed that ANXA6 induces gemcitabine resistance by inhibiting EGFR ubiquitination and degradation, which is consistent with previous studies illustrating that EGFR mRNA and/or protein expression is associated with drug resistance. In the previous study, ANXA2 acted as a molecular switch for EGFR activation, which could interact with EGFR in the same protein complex, thus suggesting the interaction of ANXA2 with EGFR [30]. EGFR overexpression was an independent prognostic factor in pancreatic cancer patients receiving gemcitabine-based adjuvant chemotherapy [31]. ANXA6 has been found in EGFR-containing protein complexes and regulates the EGFR/Ras pathway [32]. It showed that reduced expression of ANXA6 both promoted the internalization and degradation of activated EGFR and sensitized TNBC cells to EGFR TKIs [33–35]. However, this finding was not replicated in human squamous epithelial cells. Elevated ANXA6 levels enhanced the TKI-mediated inhibition of growth, migration, and invasion in EGFR-overexpressing human squamous epithelial carcinoma [36]. Thus, the effects of ANXA6 may be tumor type-specific. Although EGFR inhibitors have no effect against TNBC, the co-delivery of chemotherapeutic drugs and anti-EGFR antibodies using nanoparticles could not only enhance the EGFR-TKI’s efficacy, but also overcome the chemotherapy resistance in TNBC treatment [37, 38], thereby providing a new treatment strategy for this malignancy.

Several studies have shown that exosomes can confer resistance to therapy-sensitive tumor cells by transmitting their cargos. Despite our growing understanding of the importance of and complexity of cancer exosomes and chemoresistance, there is no consensus on dependable exosome isolation protocols at present. According to the worldwide survey from the international society for extracellular vesicles [39] and the minimal information for studies of extracellular vesicles [40], differential ultracentrifugation is the most commonly used technique and the gold standard method for primary exosomes separation and concentration. However, we used a commercially available kit (ExoQuick) to isolate exosomes in our experiments according to high-quality studies [41, 42]. It has been previously shown that not only exosomes, but also protein complexes and other non-vesicular structures are precipitated with this method [19]. Therefore, the purity of the isolated exosomes may affect the interpretation of our results. In order to overcome this disadvantage and make the results reliable, we provided several data for improvement. The shape and size of isolated exosomes were confirmed by transmission electron microscopy and NanoSight tracking analysis. Western blotting showed that these isolated exosomes were enriched in the exosomal markers CD9, CD63, and CD81, and were almost absent in the non-vesicular fraction marker fibronectin, indicating that only a very small amount of non-vesicular structures were precipitated with this method. Interfering exosome integrity by methyl β-cyclodextrin determined the loss of CD9, CD63, and CD81. We reduced exosome production through pharmacological inhibition with GW4869. The culture medium from GW4869-treated MDA-231-R cells failed to induce gemcitabine resistance to recipient cells, indicating the critical role of exosomes for the transfer of resistance. The experimental results above demonstrated that protein complexes and non-vesicular structures, even if they were precipitated with this ExoQuick kit, contributed a minimal effect on the results and it was the cell-derived exosomes we defined here playing the key role in gemcitabine resistance.

In conclusion, understanding how those chemoresistant cells-derived exosomes modify chemosensitive cells could lead to reducing tumor cell resistance and then improving chemotherapy efficacy in patients with metastatic TNBC. In the present study, we revealed that exosomal ANXA6 derived from chemoresistant cancer cells induced gemcitabine resistance by inhibiting the ubiquitination and degradation of EGFR. This biological interaction between exosomal ANXA6 and EGFR in TNBC was mirrored by statistical evidence that serum levels of exosomal ANXA6 might be a potential predictor of therapeutic response. We envision that blocking the function of exosomal ANXA6 or EGFR might be used as an alternative treatment for chemoresistant mTNBC in the future. The target may provide precise medical care and improve patients’ survival. Therefore, the significance of exosomal ANXA6 and EGFR for TNBC chemoresistance merits further investigation.

## Supplementary information

Supplementary Figure 1

Supplementary Figure 2

Supplementary Figure 3

Supplementary Figure 4

Supplementary Table 1

Supplementary Table 2

Supplementary material-Figure legends

## Data Availability

All data generated or analyzed during this study are included in this published article.
