# Supplementary figures and images for: Exosomal annexin A6 induces gemcitabine resistance by inhibiting ubiquitination and degradation of EGFR in triple-negative breast cancer
Source: Cell Death Dis. 2021 Jul 8;12(7):684. doi: 10.1038/s41419-021-03963-7 (PMC8266800; doi:10.1038/s41419-021-03963-7)

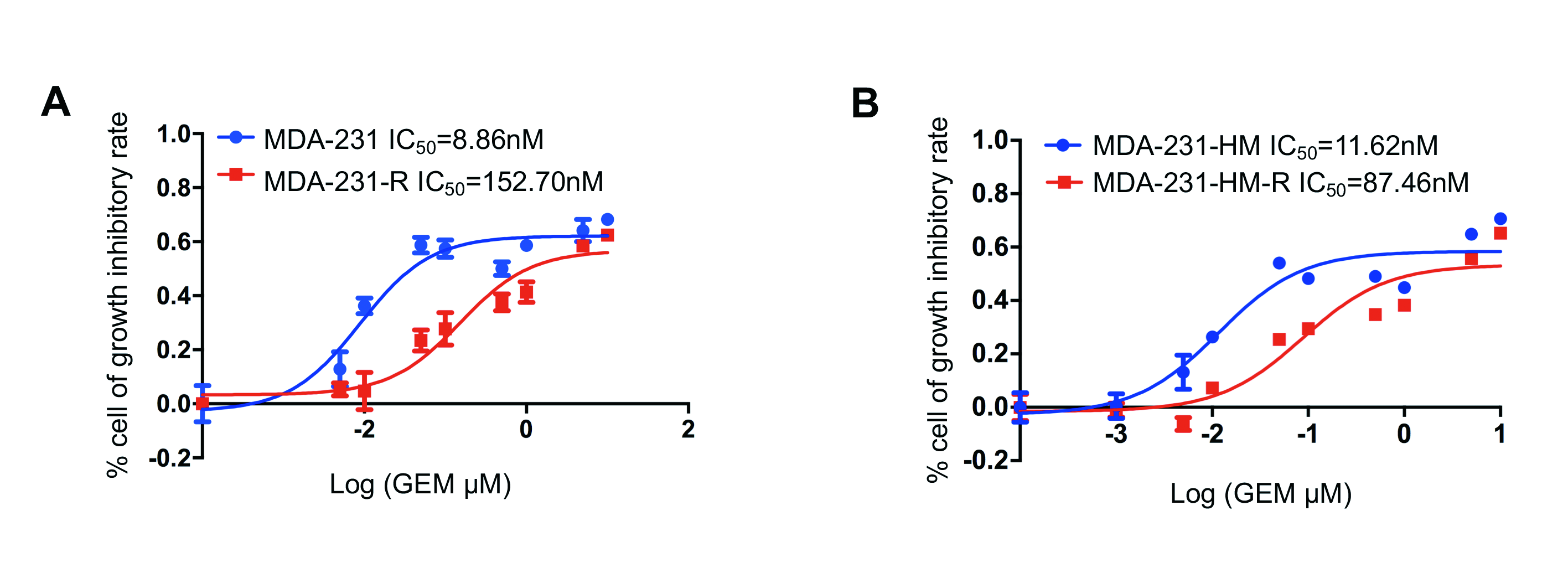

Supplement: Supplementary file 1 — Supplementary Figure 1 [file 41419_2021_3963_MOESM1_ESM.tif]

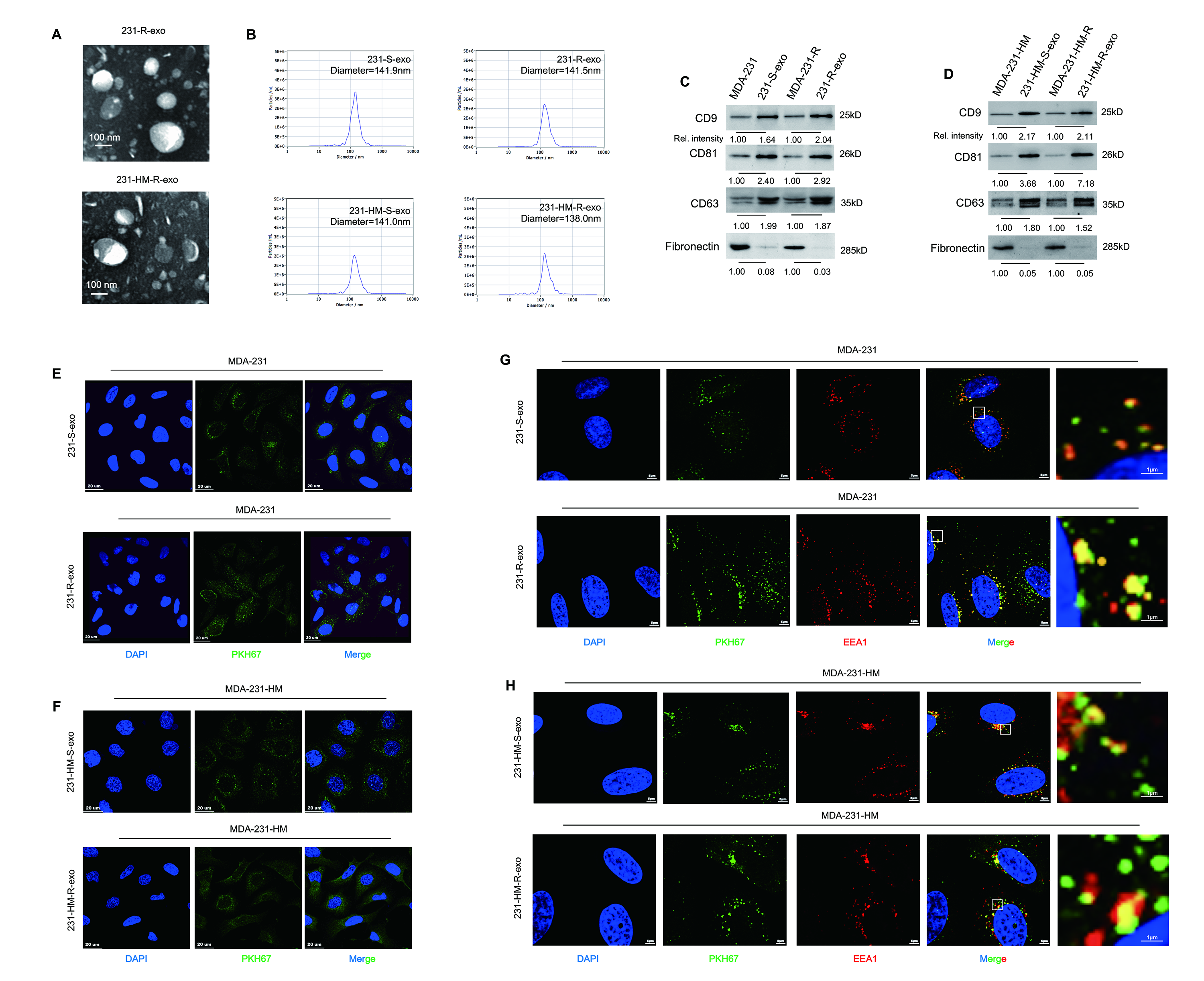

Supplement: Supplementary file 2 — Supplementary Figure 2 [file 41419_2021_3963_MOESM2_ESM.tif]

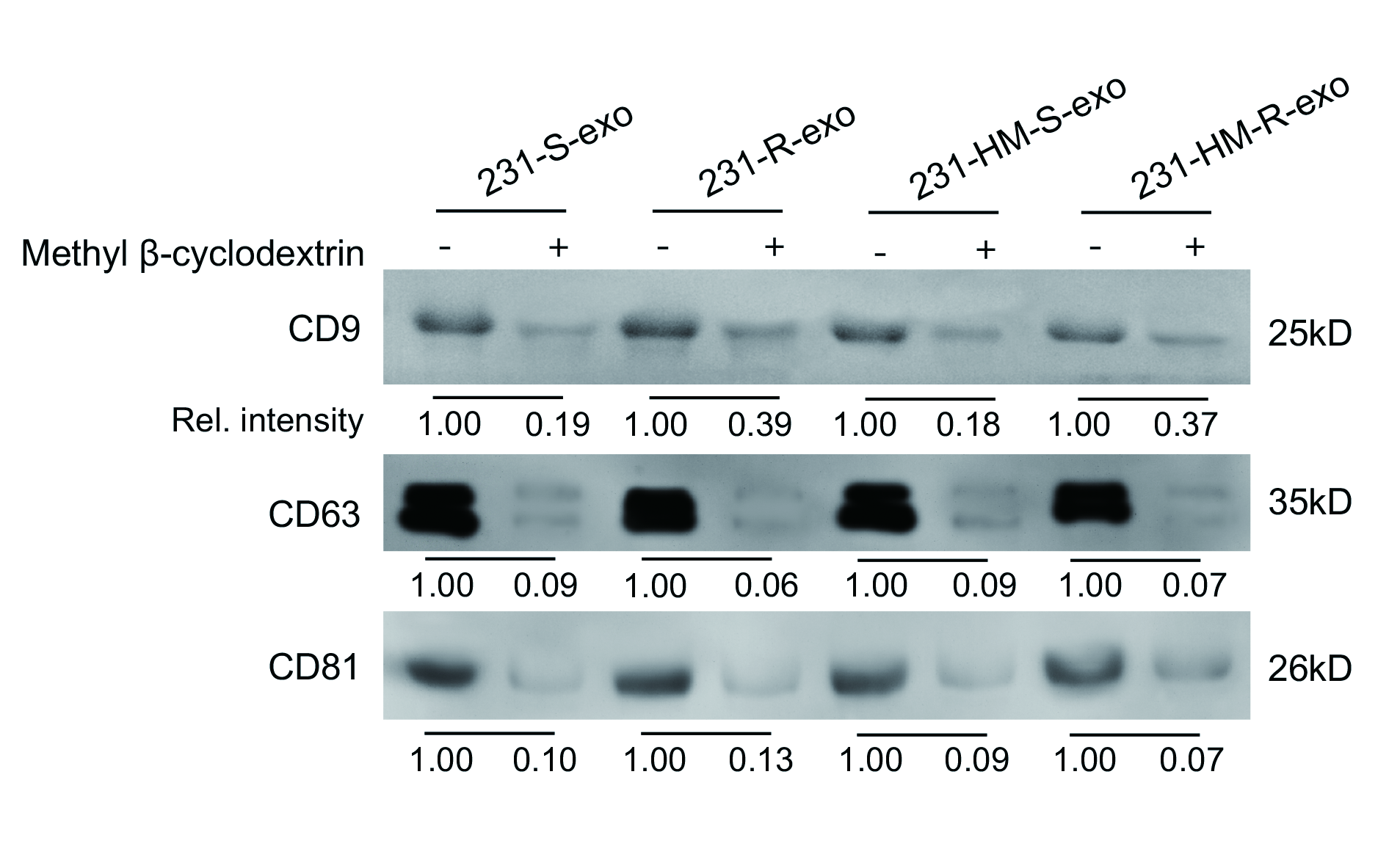

Supplement: Supplementary file 3 — Supplementary Figure 3 [file 41419_2021_3963_MOESM3_ESM.tif]

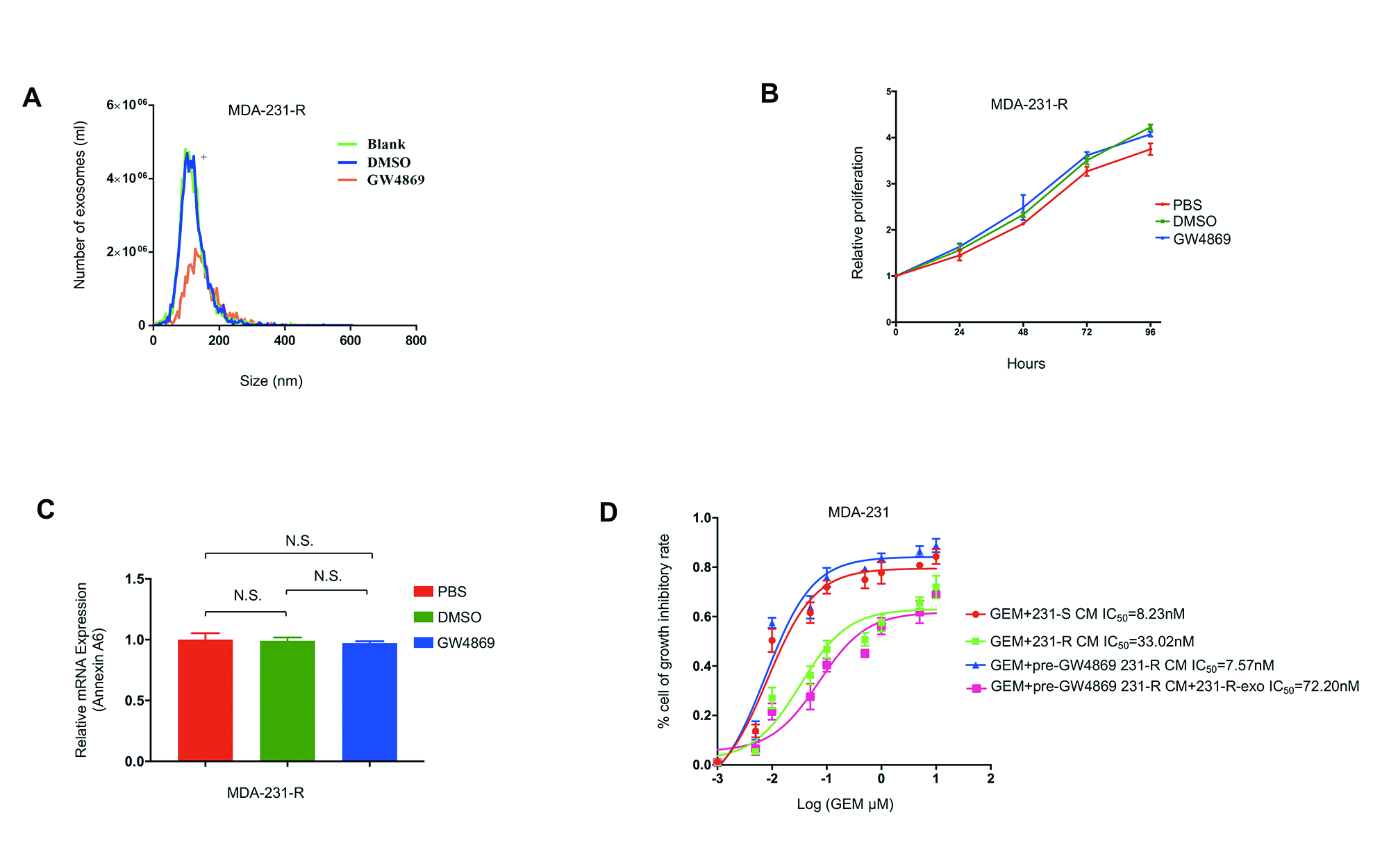

Supplement: Supplementary file 4 — Supplementary Figure 4 [file 41419_2021_3963_MOESM4_ESM.tif]
