# Supplementary material for: Exosomal annexin A6 induces gemcitabine resistance by inhibiting ubiquitination and degradation of EGFR in triple-negative breast cancer
Source: Cell Death Dis. 2021 Jul 8;12(7):684. doi: 10.1038/s41419-021-03963-7 (PMC8266800; doi:10.1038/s41419-021-03963-7)
Supplement: Supplementary file 5 — Supplementary Table 1 [file 41419_2021_3963_MOESM5_ESM.docx]

**Supplementary Table 1 Patient** **Characteristics at baseline (*N* =81)**

| Characteristics | Pts (*N*=81) | |
| --- | --- | --- |
|  | *N* | % |
| Age (Median, range) | 52, 24-80 | |
| < 40 years | 74 | 91.36 |
| ≥ 40 years | 7 | 8.64 |
| Pathological tumor size |  |  |
| 0 to ≤2 cm | 26 | 32.10 |
| 2 to ≤5 cm | 50 | 61.73 |
| > 5 cm | 5 | 6.17 |
| Nodal status |  |  |
| 0 positive nodes | 45 | 55.56 |
| 1–3 positive nodes | 19 | 23.46 |
| ≥ 4 positive nodes | 17 | 20.99 |
| Prior Adjuvant Chemotherapy |  |  |
| Anthracyclines | 1 | 1.23 |
| Taxanes | 9 | 11.11 |
| Both | 59 | 72.84 |
| Unkonwn | 12 | 14.81 |
