# Supplementary material for: Exosomal annexin A6 induces gemcitabine resistance by inhibiting ubiquitination and degradation of EGFR in triple-negative breast cancer
Source: Cell Death Dis. 2021 Jul 8;12(7):684. doi: 10.1038/s41419-021-03963-7 (PMC8266800; doi:10.1038/s41419-021-03963-7)
Supplement: Supplementary file 6 — Supplementary Table 2 [file 41419_2021_3963_MOESM6_ESM.docx]

**Supplementary Table 2 Patient** **Characteristics at baseline (*N* =21)**

| Characteristics | Sensitive Pts (*N*=14) | | Resistant Pts (*N*=7) | |
| --- | --- | --- | --- | --- |
|  | *N* | % | *N* | % |
| Age (Median, range) | 49，37-67 | | 49, 35-65 | |
| < 40 years | 1 | 7.14 | 3 | 42.86 |
| ≥ 40 years | 13 | 92.86 | 4 | 57.14 |
| Menstruation status |  |  |  |  |
| Post-menopausal | 7 | 50.00 | 3 | 42.86 |
| Pre-menopausal | 7 | 50.00 | 4 | 57.14 |
| ECOG performance status |  |  |  |  |
| 0 | 0 | 0.00 | 1 | 14.29 |
| ≥1 | 14 | 100.00 | 6 | 85.71 |
| Number of metastatic sites |  |  |  |  |
| <3 | 11 | 78.57 | 5 | 71.43 |
| ≥3 | 3 | 21.43 | 2 | 28.57 |
| Metastatic sites |  |  |  |  |
| Lymph nodes | 10 | 71.43 | 4 | 57.14 |
| Liver | 3 | 21.43 | 2 | 28.57 |
| Bone | 3 | 21.43 | 3 | 42.86 |
| Lung | 6 | 42.86 | 4 | 57.14 |
| Chest wall | 3 | 21.43 | 1 | 14.29 |
| Pleura | 1 | 7.14 | 1 | 14.29 |
| Others | 0 | 0.00 | 1 | 14.29 |
| Visceral metastasis |  |  |  |  |
| Yes | 9 | 64.29 | 5 | 71.43 |
| No | 5 | 35.71 | 2 | 28.57 |
| Disease-free interval |  |  |  |  |
| > 12 months | 12 | 85.71 | 4 | 57.14 |
| ≤ 12 months | 2 | 14.29 | 3 | 42.86 |
| Prior Adjuvant Chemotherapy |  |  |  |  |
| Anthracyclines | 1 | 7.14 | 0 | 0.00 |
| Taxanes | 2 | 14.29 | 0 | 0.00 |
| Both | 10 | 71.43 | 6 | 85.71 |
| De novo disease | 1 | 7.14 | 1 | 14.29 |
| Chemotherapy combined with gemcitabine at first-line |  |  |  |  |
| Cisplatin | 13 | 92.86 | 5 | 71.43 |
| Carbopaltin | 1 | 7.14 | 2 | 28.57 |
