# Supplementary material for: Exosomal annexin A6 induces gemcitabine resistance by inhibiting ubiquitination and degradation of EGFR in triple-negative breast cancer
Source: Cell Death Dis. 2021 Jul 8;12(7):684. doi: 10.1038/s41419-021-03963-7 (PMC8266800; doi:10.1038/s41419-021-03963-7)
Supplement: Supplementary file 7 — Supplementary material-Figure legends [file 41419_2021_3963_MOESM7_ESM.docx]

**Supplementary Figure 1. Establishment of gemcitabine-resistant cell lines. A–B** The IC_50_ for gemcitabine was determined using the CCK-8 assay. The results illustrated that after incubation with gemcitabine for 72 h, MDA-231-R cells were 17.2-fold more resistant to the drug than MDA-231 cells. Similarly, MDA-231-HM-R cells were 7.5-fold more resistant to gemcitabine than MDA-231-HM cells. All experiments were repeated three times, and the representative results are presented.

**Supplementary Figure 2. Identification of exosomes and their internalization. A** The representative transmission electron microscopy image of MDA-231-R and MDA-231-HM-R cell-derived exosomes (231-R-exo and 231-HM-R-exo). **B** Exosomes are nano-sized vesicles of approximately 100–150 nm in diameter as determined via NanoSight tracking analysis. **C–D** Expression of the exosomal markers CD9, CD81, CD63 and the non-vesicular fraction marker fibronectin in MDA-231 and MDA-231-HM cells and their derived exosomes as assessed using western blotting**. E-H** PKH67 staining and colocalization analysis in MDA-231 cells treated with sensitive cell-derived exosomes (231-S-exo), or resistant cell-derived exosomes (231-R-exo), and MDA-231-HM cells treated with sensitive cell-derived exosomes (231-HM-S-exo )or resistant cell-derived exosomes (231-HM-R-exo) for 24 h. PKH67-labeled exosomes (green) were taken up and internalized in the cytoplasm of MDA-231 and MDA-231-HM cells **(E-F)**. PKH67-labeled exosomes (green) partially colocalized with the early endosomes marked with EEA1 (red) in the cytoplasm of MDA-231 and MDA-231-HM cells **(G-H)**. All experiments were repeated three times, and the representative results are presented.

**Supplementary Figure 3. Methyl β-cyclodextrin interfered with exosomal integrity, as determined by the loss of exosomal markers.** Triple-negative breast cancer cells were incubated with 10 mM methyl β-cyclodextrin. Total medium was subjected to exosomal extraction, and the pellet was immunoblotted for CD9, CD63, and CD81.

**Supplementary Figure 4. Exosomes are necessary for gemcitabine resistance. A** NanoSight tracking analysis of the size distributions and number of exosomes from MDA-231-R cells subjected to the indicated treatments (blank, DMSO, or 10 μM GW4869) for 24 h. **B** CCK-8 assay of MDA-231-R cells treated with PBS, DMSO, or GW4869 (10 μM) for the indicated times (n = 3). **C** qRT-PCR analysis of annexin A6 levels in MDA-231-R cells treated with PBS, DMSO or GW4869 (10 μM) for 72 h (n = 3). **D** MDA-231 cells were pre-treated with culture medium (CM) from different sources for 48 h and then subjected to the CCK-8 assay following gemcitabine treatment at different concentrations (0.001, 0.005, 0.01, 0.05, 0.1, 0.5, 1.5, and 10 µM) for 48 h (n = 3). All the experiments were repeated three times, and the representative ones of those results are presented. Quantitative data are presented as the mean ± SD of triplicate experiments.
